# Glycolysis related gene expression signature in predicting prognosis of laryngeal squamous cell carcinoma

**DOI:** 10.1080/21655979.2021.1980177

**Published:** 2021-10-29

**Authors:** Hui-Ching Lau, Yujie Shen, Qiang Huang, Hui-Ying Huang, Liang Zhou

**Affiliations:** a Department of Otorhinolaryngology, Eye & ENT Hospital, Fudan University, Shanghai, China; b Shanghai Key Clinical Disciplines of Otorhinolaryngology, Shanghai, PR China

**Keywords:** Laryngeal squamous cell carcinoma, TCGA, GEO, prognosis, gene signature

## Abstract

Researches have suggested that aerobic glycolysis can reflect the development and progression of most carcinomas. We aimed to investigate whether glycolysis-related genes (GRGs) are associated with overall survival in laryngeal squamous cell carcinoma (LSCC). Here, we identified differentially expressed GRGs in TCGA dataset and microarray sample of GSE27020 from GEO database. A set of two glycolytic gene signatures, including *DDIT4* and *PLOD2* was screened through Cox and Lasso regression. The risk score was calculated using the gene expression of the two GRGs. The high-risk group presented a poor prognosis through Kaplan–Meier method. The ROC curve indicated good prediction performance in survival based on the validation of four cohorts. Univariate and multivariate Cox regression analyses suggested that two-gene signature could be an independent risk factor in LSCC. A total of 17 LSCC patients were enrolled to clarify the genetic expression through using reverse transcription-polymerase chain reaction (RT-PCR). A visualized nomogram was then constructed to predict 1-, 3-, and 5-year overall survival. Taken together, two novel glycolytic gene signatures were discovered and validated, providing a potential therapeutic and overall survival (OS)-prediction biomarker for LSCC.

## GRAPHICAL ABSTRACT

**Figure:**
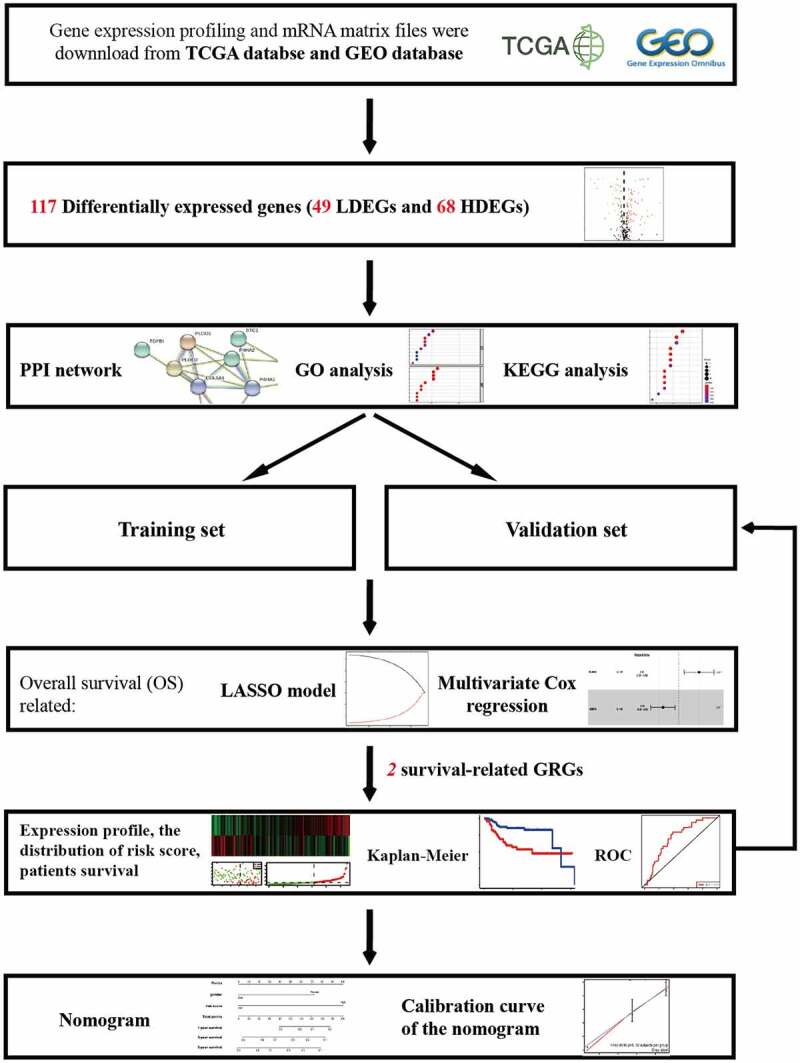

## 1. Introduction

Among all malignant tumors, the morbidity rate of head and neck carcinoma (HNC) ranks seventh [1]. Laryngeal squamous cell carcinoma (LSCC) is one of the most common cancers of the HNC in China, with estimated crude incidence and mortality rate to be at 1.84/100,000 and 1.00/100,000, respectively [2]. LSCC is male predominant with incident cases and deaths approximately seven times higher than that in women [3]. Despite the rapid improvements in diagnostic and treatment methods for the last four decades, a lack of prominent biomarkers means progress in early diagnosis remains slow.

The changed states of metabolic reprogramming, such as glucose metabolism [4], lipid metabolism [5], biological oxidation, and iron metabolism have been mentioned as important hallmarks of cancer. Warburg effect, noticed in the 1920s, revealed that malignant carcinoma cells prefer sourcing glucose through aerobic glycolysis even under conditions where oxygen is abundant, unlike the usual carbohydrate metabolism within a normal cell. The tumor microenvironment (TME) could be reconstructed by enhancing the tolerance of cellular hypoxia, acidizing the cellular matrix, and accumulating immune cells and several inflammatory factors to propagate carcinoma adaptation and help maintain their survival. Mounting researches have also revealed that glycolysis processes have been associated with the development and progression of most carcinomas such as hepatocellular carcinoma [6], lung carcinoma [7], and colorectal carcinoma [8]. The unveiling of mechanisms behind glycolysis gene in carcinogenesis covering phenotypes that boost malignant cell proliferation, invasion, and metastasis [9], immune evasion [10], stem-like cell survival, epithelial-mesenchymal transition [11], and chemotherapy resistance [12] could provide us with new insights into therapeutic opportunities, and prognostic assessments.

To our knowledge, this study may be the first one to identify and comprehensively analyze prognostic GRGs for the prediction of survival in LSCC patients based on the public TCGA database and GEO database. Although solid evidence have identified valuable glycolytic genes in HNC [13], the component of glycolysis and its function in LSCC remains rarely studied and unclear. Therefore, we attempted to profile differential glycolysis-related genes in laryngeal squamous cell carcinoma to construct a nomogram that can contribute toward short- to long-term prognosis. Our results could become an effective predictor and be of value clinically.

## 2. Materials and methods

### 2.1. Acquisition of clinical data and gene expression

RNA-sequence data and clinicopathologic information were downloaded from The Cancer Genome Atlas (TCGA) data portal (https://portal.gdc.cancer.gov/) [14]. A total of 12 normal laryngeal tissues and 111 laryngeal carcinoma tissues were enrolled [15]. The clinical information of TCGA set are shown in Supplementary Table 1. Complete RNA sequences, their corresponding follow-up time, and overall survival (OS) were included. All data were obtained from open sources, thus approval from Ethics Committee was not necessary. All samples strictly followed the access principle and publication guidelines of the TCGA database. The transcription profiling of mRNA matrix files in GSE27020 was downloaded from the GEO database (https://www.ncbi.nlm.nih.gov/geo/)). A total of 109 laryngeal carcinoma samples with clinical information and disease-free survival (DFS) were used to validate the prognosis signature. The expression signatures of GRGs were derived from the Molecular Signatures Database (https://www.gsea-msigdb.org/gsea/msigdb/index.jsp [16] [17], of which GO_GLYCOLYSIS_PROCESS, KEGG_GLYCOLYSIS_GLYCONEOGENENSIS, REACTOME_GLYCOLYSIS, BIOCARTA_GLYCOLYSIS_PATHWAY, HALLMARK_GLYCOLYSIS_GLUCONEOGENESIS pathways were included. A total of 288 potential glycolysis-related genes were found from the former database.

### 2.2. Bioinformatic differentially expressed GRGs analysis

Adjusted *p*-value was calculated into false discovery rate (FDR). FDR < 0.05 and |LogFold change (FC)| ≥0.5 are considered to be of significant differences. Differentially expressed GRGs were visualized in heatmap and volcano map via R software (version: x64 3.6.1) [18].

### 2.3. Pathway enrichment analysis

Gene Ontology (GO) and Kyoto Encyclopedia of Genes and Genomes (KEGG) pathway analyses were used to identify potential biological processes (BP), cellular components (CC), and molecular functions (MF) of differentially expressed GRGs [19–21]. The differentially expressed GRGs were categorized into uprising and downregulating GRGs groups. Significant relevant signal pathways were identified using R software. The categorized groups were analyzed and presented using bar graphs and bubble graphs. The potential interaction between differentially expressed GRGs was discovered in STRING database (https://string-db.org) with a minimum required interaction score≥0.4 [22]. After casting out the discrete value, the differentially expressed GRGs of the network were screened for further study.

### 2.4. Identification of prognosis signature based on differentially expressed GRGs

The differentially expressed GRGs were validated with univariate Cox regression proportional hazard regression model. GRGs with *p*-value less than 0.05 were considered to be significantly correlated with survival and were included in subsequent analyses. Lasso regression analysis was used to reduce the numbers of GRGs and were presented using cvfit plot and lambda plot. The cvfit plot showed the lowest value of the curve as penalized value. The lambda plot was used to screen prognosis-related variables based on the penalized value. Multivariate Cox regression analysis were applied to construct a prognosis signature. A prognostic gene signature was then built on a linear combination of the regression coefficient derived from the Lasso Cox regression model coefficients (*β*) multiplied with its mRNA expression level. The model function is as follows: Risk score = *β_1_* * expression (gene A) + *β_2_* * expression (gene B) + *β_3_* * expression (gene C) [23–25]. The median value in risk score would be a cutoff point conducted to segregate TCGA dataset and GEO dataset into high- and low-risk groups, respectively. Risk plot, survStat plot, heatmap plot, and receiver operating characteristic (ROC) plot were used to demonstrate the efficacy of this cutoff point in the training set. The survStat plot was used to demonstrate the distribution of survival status in the long term as the risk score elevated. The testing set, GSE27020 set, and the TCGA entire set were then validated with the same analyses.

### 2.5. Identification of independent prognostic parameters in LSCC

To validate and identify the independent prognostic parameters, risk score, age, gender, TNM staging, pathological grade of TCGA entire set were enrolled in univariate Cox regression and multivariate regression. The TNM staging was constituted by primary tumor site (T category), regional lymph node involvement (N category) and the presence of distant metastatic spread (M category). *P*-value <0.05 was considered statistically significant. Parameters with *p-*value lower than 0.05 on the univariate analysis were further included in multivariate Cox regression analysis to identify independent risk factors.

### 2.6. Validation under reverse transcription-polymerase chain reaction (RT-PCR)

A total of 17 male patients with laryngeal carcinoma were enrolled in this study from November 2020 to December 2020. The clinical information was shown in Supplementary Table 2. Written informed consent was acquired from all participants. Ethical approval was granted by the Ethical Committees of Eye and ENT Hospital, Fudan University. Information of patients was anonymized prior to analysis. The inclusion criteria in LSCC group were as follows: (1) confirmed pathologic squamous cell carcinoma, (2) complete clinical and laboratory data before operation. The exclusion criteria were as follows (1) patients that received chemotherapy and radiotherapy before operation, (2) possessed other carcinomas, (3) possessed chronic inflammation, and (4) unable to obtain paired tissues. Carcinoma tissues paired with adjacent tissues were collected in Trizol reagent (Invitrogen, Thermo Fisher Scientific, USA). Each reagent contained ≤1000 ng mRNA and was reverse transcribed in TAKARA 99420 kit. A total of 1 μL cDNA was amplified in triplicate in 10 μL reactions containing 2× QuantiNova SYBR Green PCR Master Mix (QIAGEN, Germany), and 0.2 μM of paired primer in a 96-well optical PCR plate. Reverse transcription-polymerase chain reaction (RT-PCR) was performed with the following primers, **GAPDH** (NM_002046.7): GAPDH forward primer: 5ʹGATGCCCCCATGTTCGTCAT-3ʹ; GAPDH reverse primer: 5ʹ-TAAGCAGTTGGTGGTGCAGG-3ʹ; **PLOD2** (NM_182943.3): PLOD2 forward primer: 5ʹ-TCGAGCATCCCCACAGATAAAT-3ʹ, PLOD2 reverse primer: 5ʹ-ATCTCACTTTCTGGCCCCCT-3ʹ; **DDIT4** (NM_019058.4): DDIT4 forward primer: 5ʹ- CTTCTCGTCGTCGTCCACCT-3ʹ, DDIT4 reverse primer: 5ʹ-CATCCAGGTAAGCCGTGTCTTC-3ʹ. Amplification and detection of DNA were performed with the ABI 7500 Real-Time PCR System (Applied Biosystems, USA) under the following reaction conditions: 5 min at 95°C, followed by 40 cycles of denaturation at 95°C for 10 s and 60°C for 34 s. The cycle threshold (Ct) values were used to analyze and validate the number of genes in each sample. GAPDH was used as internal control and the -ΔCT method was performed. Data Analysis was performed with SPSS 20.0 software (SPSS Inc, Chicago, IL) and 8th edition GraphPad Prism software [26,27]. Paired *t*-test was used to compare the biodiversity between carcinoma and adjacent normal groups.

### 2.7. Predictive nomogram construction and validation

Based on “rms’ package of R software [18,28], the independent risk factors such as gender, and risk score were used to evaluate the performance of nomogram for predicting 1-, 3-, 5-year OS of LSCC. Calibration curve was used to testify the efficacy of nomogram

### 2.8. Statistical analysis

R software (version: x64 3.6.1) was used to perform statistical analysis [18]. Categorized variables were analyzed by chi-square distribution and Fisher’s exact test. Continuous variables were analyzed through Student’s *t*-test. Multiple groups of continuous variables were analyzed by ANOVA. Univariate and multivariate Cox regression analyses were performed to evaluate prognosis. The hazard ratio (HR) and 95% confidence interval (CI) were calculated to assess the survival-related GRGs.

## 3. Results

Understanding whether aerobic glycolysis is associated with the prognosis of LSCC may be a valuable contribution to LSCC treatments. This study aimed to investigate GRGs based on TCGA and GEO databases and screened out prognosis-based glycolysis-related genes. The genetic validation in LSCC was carried out and a nomogram was constructed for further clinical practices.

### 3.1. Patient characteristics

**Figure 1.**
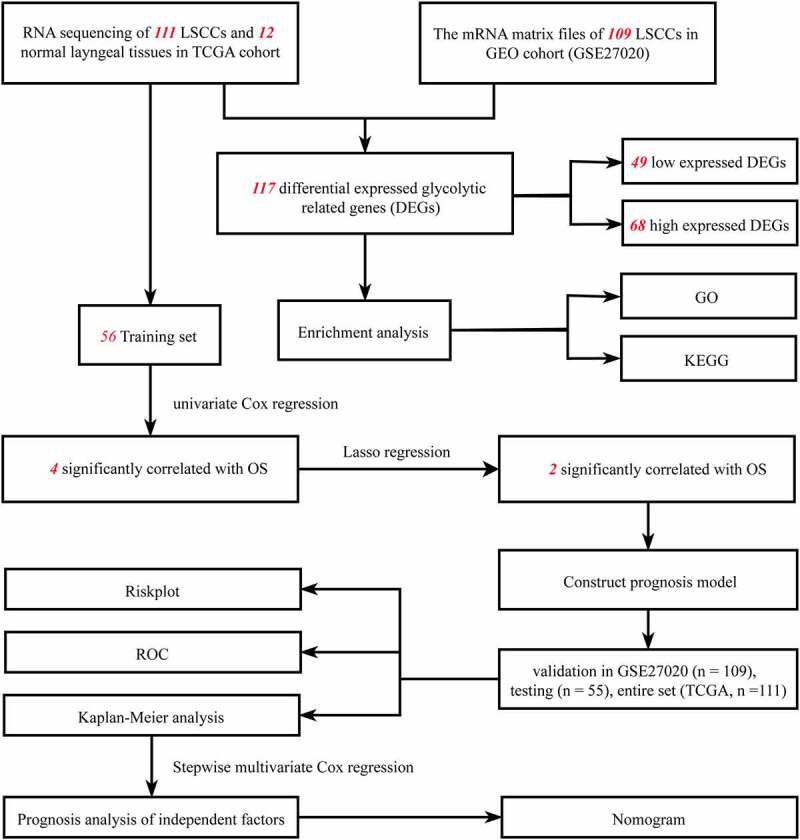
Flowchart of this study

**Figure 2.**
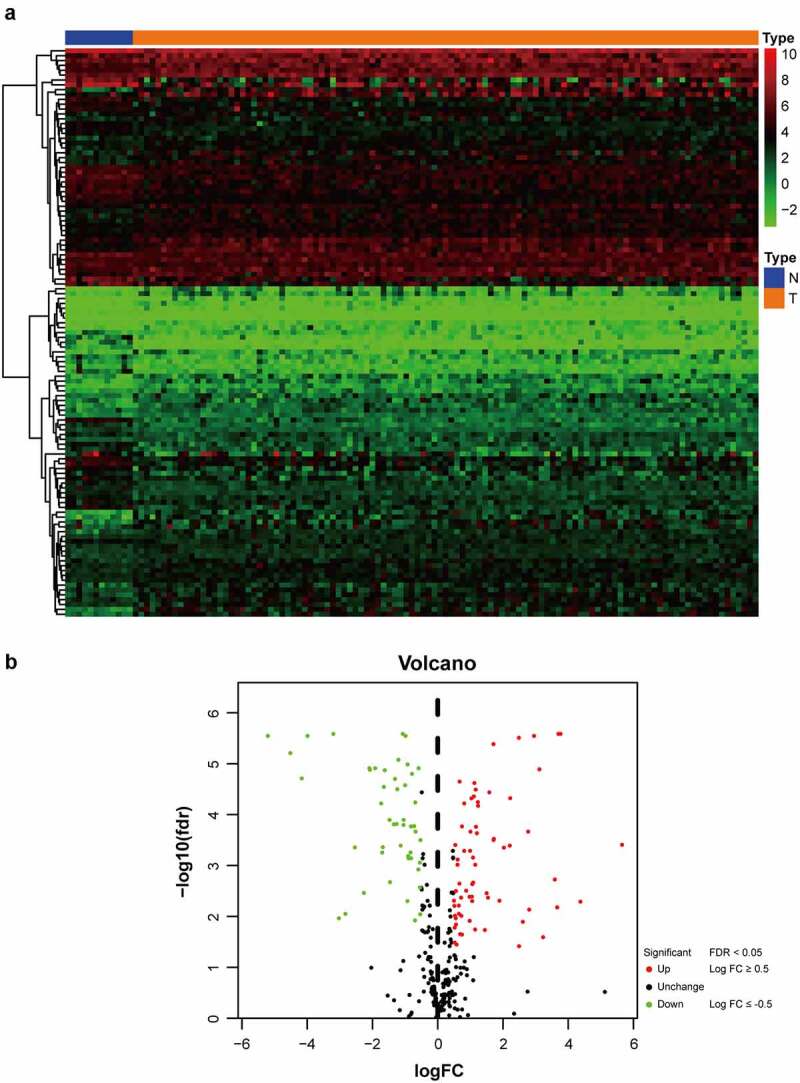
The distribution of DEGs of glycolysis in TCGA dataset. (a) Heatmap of the 117 DEGs was screened out in the 12 normal tissue and 111 carcinomas through GSEA. (b) Volcano plot of the differentially expressed GRGs between tumor specimens and normal laryngeal specimens. The *Y*-axis represents the adjusted FDR, and the *X*-axis represents the value of the log fold change (Log FC). Aberrantly expressed GRGs were calculated using the R package. Red dots represent upregulated GRGs in the tumor specimens, whereas green dots indicate downregulated. Black dots presented GRGs without significant differences in these two groups

As shown in Figure 1 flow chart, the clinical features of 111 patients with LSCC and the expression data set for 24,991 mRNAs were collected from the TCGA database. Twelve normal laryngeal samples, 111 LSCC samples from the TCGA dataset, and 109 LSCC samples from the GEO dataset were extracted. One hundred and seventeen GRGs with statistical significance from TCGA dataset was selected. The standard for differential analysis was set as follows: |log FC| ≥0.5, FDR < 0.05. Results returned 49 genes that had low expression, whereas 68 genes were of high expression in LSCC, as shown in Figure 2. These GRG expressions would be further analyzed.

### 3.2. Identifying the functional analysis of variant genes

**Figure 3.**
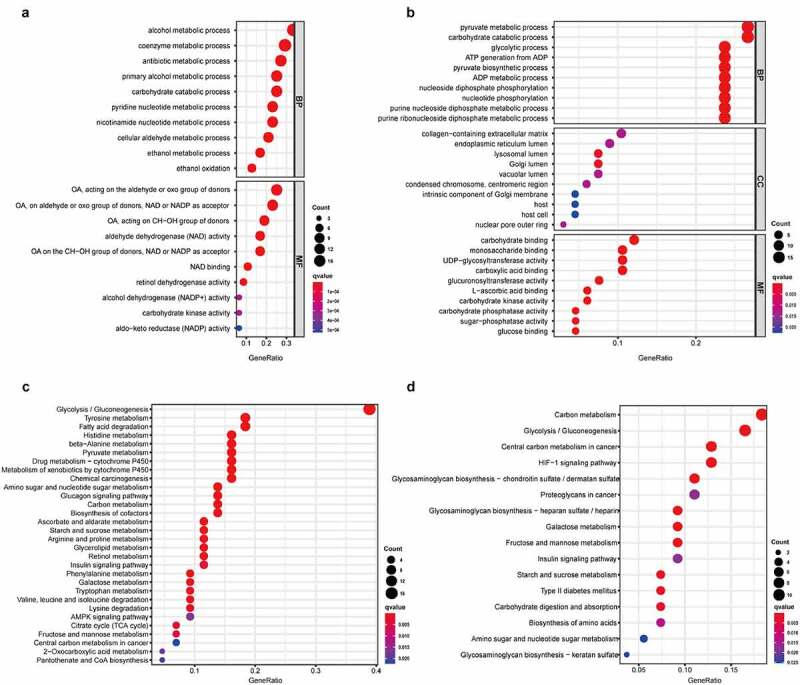
The correlation between molecular process and differential genes in LSCC. In GO analysis, 49 low expressed DEGs (a) and 68 high expressed DEGs (b) were discovered their connection to biological processes (BP), cellular components (CC) and molecular functions (MF). In KEGG analysis, 49 low expressed DEGs (c) and 68 high expressed DEGs (d) were manifested association to suspicious pathway and function. The *q*-value was deemed as an adjusted *p-*value, which <0.05 would be considered statistically significant

Gene Ontology (GO) and Kyoto Encyclopedia of Gene and Genome (KEGG) functional enrichment analyses were applied to analyze the function of genes after categorizing the low differentially expressed genes (LDEGs) and high differentially expressed genes (HDEGs). The results of KEGG analysis indicated pathway in glycolysis/gluconeogenesis were closely associated with the genes regardless of whether they were in the amplified gene group or deleted gene group. HIF-1 signaling pathway was present mainly in HDEGs, whereas the amino acid metabolism pathway was discovered to be correlated with gene LDEGs (Figure 3). GO terms such as pyruvate metabolic process, carbohydrate catabolic process, and glycolytic process were mainly enriched in the ‘biological process’ category of HDEGs. In contrast, alcohol metabolic process, coenzyme metabolic process, and antibiotic metabolic process were enriched in LDEGs. These processes are highly connected to the basic metabolism and development of TME, which might proceed to influence the progression of carcinoma.

**Figure 4.**
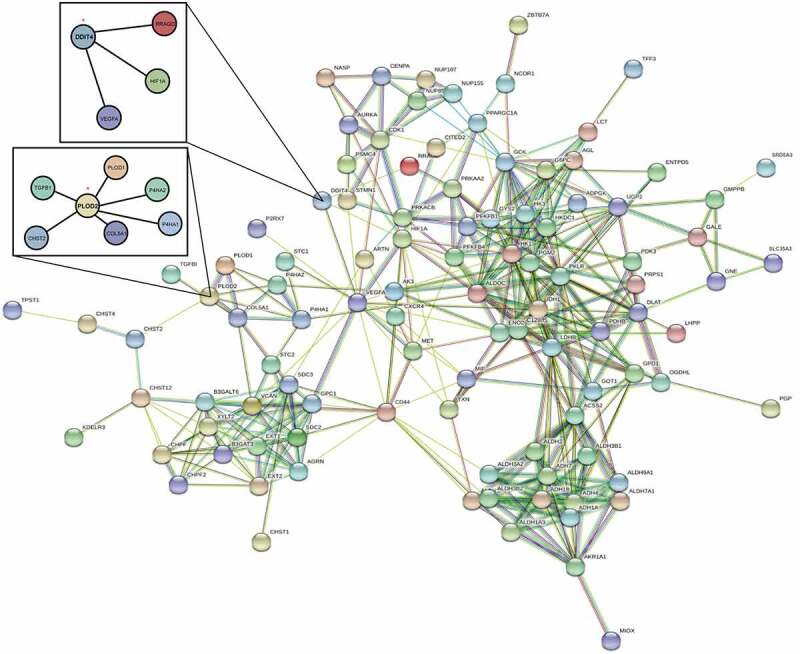
The PPI network of two differential genes and the most frequently altered neighbor genes

A PPI network of differential expressed GRGs through STRING database (Figure 4) was then constructed after removing the 14 deviated values. Based on results from the GO and KEGG analyses, the PPI network concentrates on GRGs and hypoxia-inducible factor-1 (HIF1) pathways.

### 3.3. Construction of survival‑associated glycolysis signature and validation

**Figure 5.**
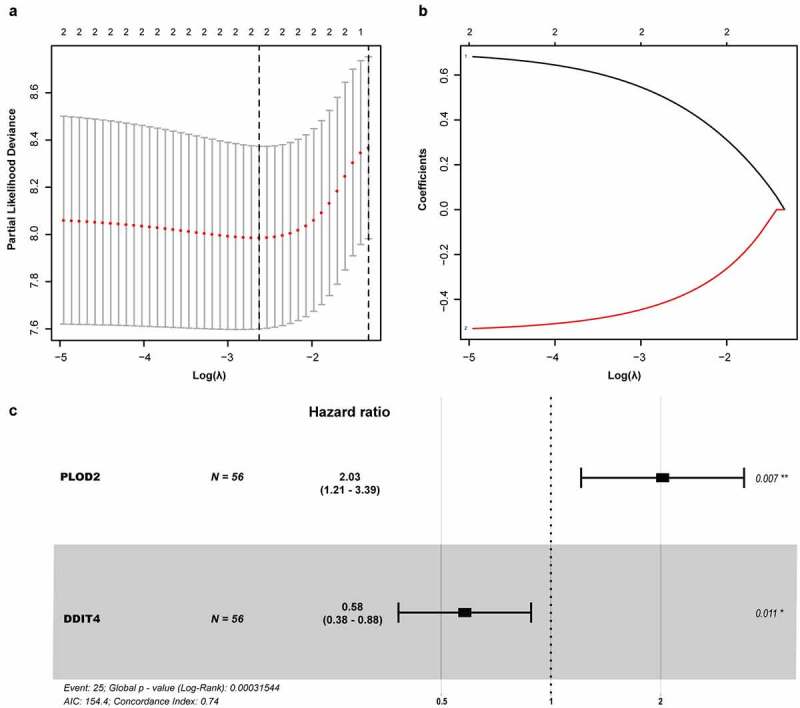
Prognostic model of the GRGs. (a and b) After screening out from univariate cox regression, the partial likelihood deviation and coefficients were calculated by LASSO. (c) The multivariate cox regression showed only *PLOD2* and *DDIT4* were enrolled. A *p*-value <0.05 would be considered statistically significant

**Figure 6.**
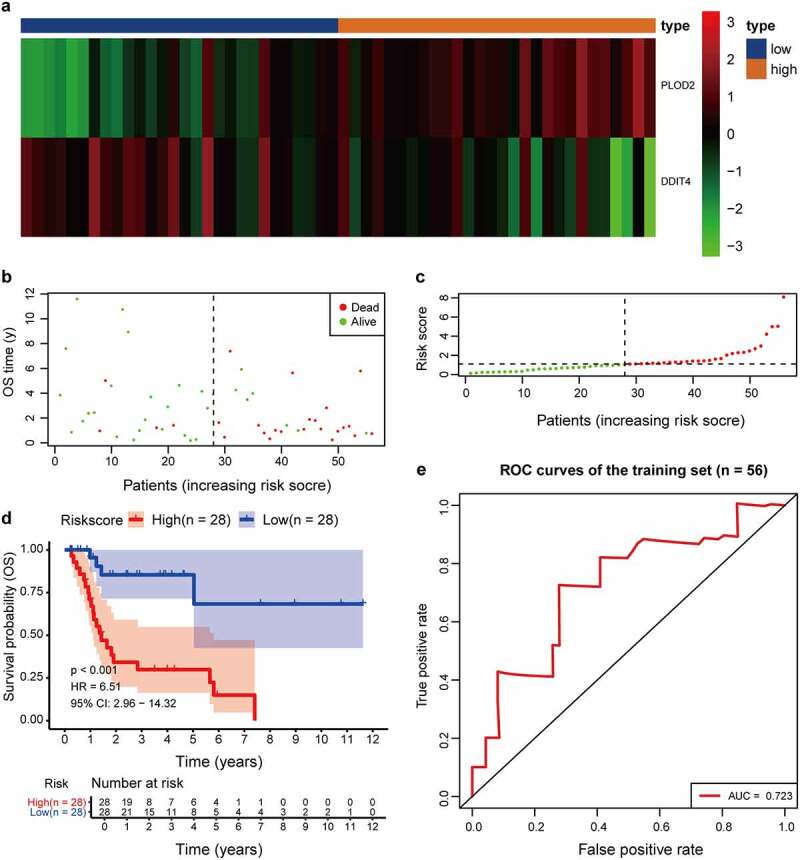
Comparison in stratified with risk signature of the two glycolysis-related hub genes in the training set. (a) *PLOD2* showed higher expression in high-risk signature (orange), whereas *DDIT4* presents higher in low-risk signature (blue). The distribution of hub genes was marked as a low proportion (green) or high proportion (red), separated by the median (vertical black line). Each point presents their connection with overall survival (b), high- and low-risk score group(c). (d) Number at risk for each stratified risk group over 12-years is processed using Kaplan–Meier method. (e) The efficacy of the risk score in predicting overall survival through ROC curve and the AUC is 0.723

A univariate Cox regression analysis of genes based on overall survival for preliminary screening in the training set was first performed (Table 1). A gene would be regarded as significantly correlated to survival if HR ≠ 1 and *p*-value <0.05. Only four genes were included: DDIT4 (*p* = 0.003), PLOD2 (*p* = 0.003), PRPS1 (*p* = 0.003); PRKACB (*p* = 0.0048). Lasso-penalized Cox regression was performed, and one risk gene and one protective gene were used to construct a predictive signature (Figure 5(a-c)) after being processed using multivariate Cox proportional regression. The risk score formula would be employed as follows: the expression level of PLOD2×0.7058+ DDIT4*-0.5438. The glycolysis-related gene *PLOD2* was found to be higher in the high-risk group, whereas DDIT4 decreased in the training set and was further validated in the testing set, GEO set, and TCGA set (Figure 6(a), Supplementary Figure S1–S3a). Patients were stratified into high- and low-risk groups based on their median risk score, which was 1.0988. The rising proportion of death events often exists in high-risk group patients (Figure 6(b,c), Supplementary Figure S1-3b-c). In Kaplan–Meier analysis, patients in the high-risk group presented worse survival outcomes, compared to the low-risk group (Figure 6(d), Supplementary Figure S1–3d). ROC curve analysis showed that the specificity and sensitivity were highest when the value of area under the curve (AUC) were 0.71 in GEO set, 0.723 in the training set, 0.77 in the testing set and 0.732 in the entire set of TCGA (Figure 6(e), Supplementary Figure S1–3e).

**Table 1.** Univariate and multivariate Cox regression of prognosis-based GRGs

	Univariate HR (95% CI)	*p*-Value	Multivariate HR (95% CI)	*p*-Value
DDIT4	0.502 (0.318–0.792)	0.003	2.025 (1.210–3.389)	0.011
PLOD2	2.049 (1.272–3.301)	0.003	0.580 (0.381–0.882)	0.007
PRPS1	5.937 (1.343–26.239)	0.018		n/a
PRKACB	2.134 (1.005–4.531)	0.048		n/a

*p* -Value <.05 presents statistically significant.

**Figure 7.**
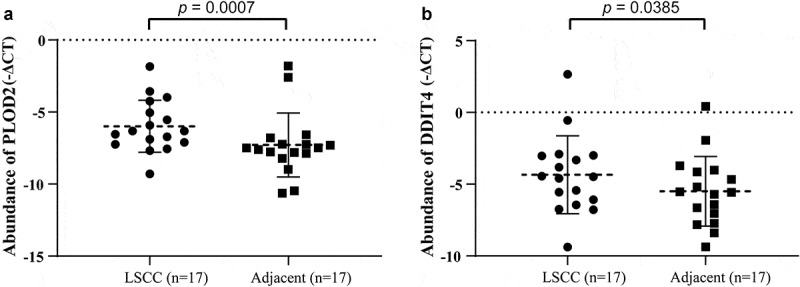
The validation of PLOD2 and DDIT4 in LSCC tissue and their adjacent tissue. (a) The PLOD2 present higher expression in LSCC, compared to their adjacent normal tissue (*p* = 0.0007). (b) The PLOD2 present higher expression in LSCC, compared to their adjacent normal tissue (*p* = 0.0385). * means *p* < 0.05; ** means *p* < 0.005

We proceeded to validate the PLOD2 and DDIT4 in 17 LSCCs and their paired adjacent normal tissue. We found that both PLOD2 and DDIT4 were enhanced in LSCC tissues, compared to adjacent tissues, which presented statistical differences consistent with our findings in a predictive model (Figure 7(a,b)).

### 3.4. Hierarchical analysis of clinical features and GRGs

We proceeded to screen out independent risk factors through the univariate and multivariate Cox proportional hazards regression analysis in TCGA set. Age, gender, TNM stage, pathological grade and risk score of combining glycolytic-related genes *PLOD2* and *DDIT4* were taken into account. Only risk score and gender were the vital and independent predictive elements to prognosis based on results from the multivariate analysis (Table 2).

**Table 2.** Univariate and multivariate Cox regression of dependent risk factors

	Univariate HR (95% CI)	*p*-Value	Multivariate HR (95% CI)	*p*-Value
Riskscore (high/low)	1.581 (1.199–2.083)	0.001	1.629 (1.090–2.436)	0.017
Age, years (>60/≤60)	0.996 (0.942–1.053)	0.9	n/a	
Gender (male/female)	0.113 (0.042–0.301)	<0.001	0.118 (0.038–0.368)	<0.001
Grade (G1-G2/ G3)	0.740 (0.321–1.707)	0.481	n/a	
Stage (I–II/III–IV)	1.036 (0.411–2.613)	0.938	n/a	

*p*-Value <.05 presents statistically significant.

### 3.5. Building predictive nomogram

**Figure 8.**
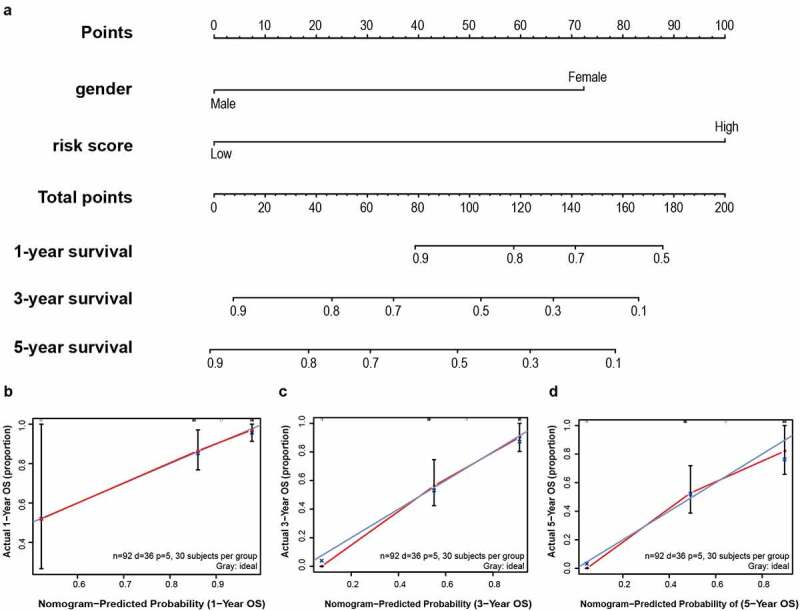
A nomogram was established for predicting 1-, 3- and 5-year OS. (a) Nomogram associated with statistically differentiated 2-mRNA signature and genders to predict short-termed to the long-termed OS in patients with LSCC. (b–d) The calibration curve of the nomogram for the prediction of 1-, 3- and 5-year OS. The blue solid line shows an ideal nomogram and the red solid line indicates the current nomogram. Two lines approach together means the nomogram performed well. Vertical lines represent 95% CI

To establish a meaningful clinical method, we created the nomogram based on the entire TCGA cohort to predict 1-, 3- and 5-year OS. The predictors of the nomogram contained gender, and risk scores (Figure 8(a)). The 45° line represents the best prediction. Calibration plots suggested that the nomogram performed well (Figure 8(b-d)).

## 4. Discussion

Laryngeal carcinoma, one of the common upper respiratory carcinomas, is correlated with risk factors such as long-term tobacco, alcohol, and also occupation exposure. China is the nation with the second-highest incidence of LSCC. Thus, attention has been focused on improving patients’ quality of life, comprehensive treatments such as radiotherapy, chemotherapy, targeted therapy, immune therapy, and great progress has been achieved. However, none of the treatments yielded an ideal 5-year survival rate, remaining at 60% without improvements in the past 10 years [29]. The metabolic process is the most important biological process in bodies to maintain homeostasis between anabolism and catabolism. As one of the hallmarks of carcinogenesis, aerobic glycolysis has been highlighted as a pervasive way of energy metabolism that is linked to many carcinomas’ biological behavior. Mounting studies had proved that glycolytic genes could be used as a prognostic biomarker. Since no studies have linked glycolytic genes with prognosis in laryngeal carcinoma, we constructed a risk score signature from GRGs to prove the prediction efficacy.

To clearly understand the function of differential gene set in a carcinoma sample when compared to normal sample, we found that regardless of LDEGs or HDEGs, glycolysis/gluconeogenesis was closely associated under GSEA analysis. Upon narrowing down which GRGs could be associated with prognosis, a new prognostic signature was built and co-considered by using microarray and RNA-sequencing data for gene expression levels or mutations. We enrolled both the KEGG dataset and GO dataset to avoid selection bias. The LSCC samples of the KEGG dataset were randomly classified into training cohort, testing cohort, and entire cohort. The training cohort was the first to have its prognostic model constructed through univariate and multivariate Cox regression. The entire cohort, and the GEO dataset then went through validating. Four glycolysis-related prognostic genes including *DDIT4*, *PLOD2*, *PRPS1*, *PRKACB* were found in the univariate Cox model, but only *DDIT4*, *PLOD2* were screened out in Lasso-penalized Cox regression and enrolled in multivariate Cox regression to determine the independent factors. We testified these two hub GRGs in most patients with laryngeal carcinoma and found them highly expressed compared to adjacent normal tissues.

DNA damage-inducible transcript 4 (DDIT4), with an alternative name of regulation in development and DNA damage response 1 (REDD1), was found in 2002 and is considered a novel HIF-responsive gene [30]. Abundant evidence had demonstrated that over-expressed DDIT4 not only becomes enriched in hypoxia or stress environment but it is also associated closely with DNA damage, inflammation, reactive oxygen species (ROS) [31], and autophagy in malignancy formation. DDIT4 was an endogenous inhibitor of the mammalian target of rapamycin C1(mTORC1) pathway through activating TSC1/2 and NF-κB pathway [32]. Due to its negative effect in Akt, DDIT4 can maintain glucose homeostasis by regulating glucose uptake and glycolysis in macrophages [33]. However, it is still controversial to deem DDIT4 as a tumor suppressor. Most malignant carcinomas such as myeloid leukemia, colon, skin, head and neck carcinoma, and breast carcinoma present high, sustained, constitutive expression of DDIT4 with poor prognosis [34,35]. However, in RAS mutant lung and pancreatic tumors, loss of DDIT4 was found to indicate poor outcome, which may result from reprogramming fatty acid oxidation and accumulating lactate and pyruvate [36]. To date, DDIT4 has been discovered to be able to interconnect mTORC1, p53, HIF, autophagy, in oxygen sensing signal pathways. More research should be conducted to investigate each of their roles as all have been proven to be associated with malignant behavior of tumors.

Recombinant procollagen lysine-2-oxoglutarate-5-dioxygenase (PLOD2), a functional homodimeric enzyme, was located at the membrane of the rough endoplasmic reticulum (RER). The function of PLOD2 could specifically hydroxylate lysine in the telopeptide of procollagens, which is essential for the biogenesis of normal mature collagen, tissue remodeling, and the stability of collagen crosslinks. Without proper regulation of the PLODs family, collagen remodeling of extracellular matrix (ECM) in TME was triggered. With collagen in solid tumor body enhanced, infiltration, invasion and distant metastasis were facilitated [37,38]. To date, several types such as lung cancer [39], breast cancer [40], colorectal cancer [41] had revealed highly expressed PLOD2 with a poor prognosis. However, only two studies had reported the mechanism of PLOD2 in LSCC. Sheng et al. had proven that rising PLOD2 in both LSCC cell line Hep-2 and hypopharyngeal squamous cell carcinoma (HPSCC) cell line Fadu could promote stem-like characteristics in cancer through validating CD44/CD133 and has a significant connection to canonical Wnt signal pathway [42]. The drug-resistance-related genes *MDR-1* and *MRP* were activated by PLOD2 which might be the reason they are connected to the prognosis. Huang et al. demonstrated that miRNA-124 also took a vital role in regulating the PLOD2 in LSCC, of which the following proliferation, migration, and invasion in vitro were suppressed [43]. Moreover, we discovered that PLOD2 and DDIT4 were regulated through HIF-1alpha in TME. Thus, we speculate that it might improve the prognosis of LSCC by inhibiting the HIF/PLOD2 or HIF/DDIT4 axes [44].

Although Liu et al. and Chen et al. have investigated the glycolysis gene signature in HNC, our study was the first to identify and comprehensively analyze prognostic GRGs for the prediction of survival in LSCC patients [45,46]. Focusing on LSCC allowed for a more comprehensive analysis, highlighting significant divergences when for different parts of HNC in terms of biological characteristics and their long-term survival rate. Comparing our models based on TCGA samples, we found our model could prove its value with higher AUC (Supplementary Figure 4). In addition, we discovered that hypoxia might be the key risk factor in the progression of LSCC, where HIF signaling pathway was found to be highly associated with both glycolytic genes *PLOD2* and *DDIT4*. Uprising hypoxia was known to interfere with the TME, especially some immune cells such as NK and CD8+ effector T cells. Considering that most LSCC patients possess cigarette and alcohol addiction, HIF-1α transcription might be a factor connecting glycolytic metabolism and immunotherapy, which is worthy of further validation. Some studies have also revealed that females with LSCC present a better prognosis than males, yet given that most LSCC cases were male-predominant, reports covering this topic are few [47,48]. However, it is interesting to notice that females with LSCC present worse health-related quality-of-life (HRQoL) compared to male population [49]. Furthermore, Aliaa Atef et al. disclosed that estrogen receptors (ER), like progesterone receptors (PR), could be risk factors in poor biological progressions of LSCC [50]. Therefore, the gender factor remains controversial, which should be validated using large female LSCC sample sizes. Our study discovered that female patients with a high-risk score of the glycolytic gene showed unsatisfactory prognosis and yielded worse prognosis than in the male population. After mining from the public database, two meaningful GRGs were narrowed down and validated, which none of the studies mentioned. This signature could act as a screening tool for those with high-risk factors in following molecular biology research and become an effective tool for physicians to predict prognosis in clinics.

Several limitations should be mentioned in this study: the majority of patients enrolled in TCGA and GEO database were Caucasian. Caution must be taken due to ethnic differences; thus, different populations need to be further studied. The small amount of validated genomics might also contribute to biases, which could affect our model’s efficacy. Moreover, the nomogram based on the TCGA database should be verified with external datasets. Therefore, expanding the sample size in different ethics and different academic facilities could be an efficient way to fix the selection bias, and large-scale experimental studies need to be conducted to elucidate the application and function of our findings in DEGs of glycolysis.

## 5. Conclusion

The prognostic glycolysis pathway genes associated with laryngeal squamous cell carcinoma were identified and used to construct a two-gene risk profile by mining the database. Higher risk parameters in LSCC indicated dissatisfactory OS, especially in the female population. The screened hub-gene might serve as a new target for further research and treatment in LSCC, which could be simply applied in clinical practice to predict short-termed and long-termed prognosis.

## Supplementary Material

Supplemental Material

## Highlights

1. Two hub genes, *DDIT4* and *PLOD2*, involved in glycolytic metabolism are correlated with short-long termed prognosis.
2. The risk score based on two glycolytic genes was calculated as an independent prognostic factor. LSCC patients with high-risk scores showed worse OS.
3. These identified genes could be potential treatments of LSCC.
