# Supplementary material for: Glycolysis related gene expression signature in predicting prognosis of laryngeal squamous cell carcinoma
Source: Bioengineered. 2021 Oct 29;12(1):8738–52. doi: 10.1080/21655979.2021.1980177 (PMC8806568; doi:10.1080/21655979.2021.1980177)
Supplement: Supplemental Material [file KBIE_A_1980177_SM4054.zip › supplementary/Legends of Supplementary Tables and Figures (1).docx]

**Legends of Supplementary Tables and Figures**

**Supplementary Table 1.** The clinical information of TCGA set.

**Supplementary Table 2.** The clinical information of enrolled 17 male patients with LSCC.

**Supplementary Figure 1.** Comparison in stratified with risk signature of the 2 glycolysis-related hub genes in GEO dataset. (A) PLOD2 show higher expression in high-risk signature (orange), whereas DDIT4 present higher in low-risk signature (blue). The distribution of hub genes was marked as low proportion (green) or high proportion (red), separated by the median (vertical black line). Each point presented their connection with disease-free survival (B), high and low risk score group(C). (D) Number at risk for each stratified risk group over 3 years is processed using Kaplan-Meier method. (E) The efficacy of risk score in predicting disease-free survival through ROC curve and the AUC is 0.71.

**Supplementary Figure 2.** Comparison in stratified with risk signature of the 2 glycolysis-related hub genes in the testing dataset. (A) PLOD2 showed higher expression in high-risk signature (orange), whereas DDIT4 presented higher in low-risk signature (blue). The distribution of hub genes was marked as a low proportion (green) or high proportion (red), separated by the median (vertical black line). Each point presented their connection with overall survival (B), high and low-risk score group(C). (D) Number at risk for each stratified risk group over 20 years is processed using Kaplan-Meier method. (E) The efficacy of risk score in predicting overall survival through ROC curve and the AUC is 0.77.

**Supplementary Figure 3.** Comparison in stratified with risk signature of the 2 glycolysis-related hub genes in TCGA dataset. (A) PLOD2 show higher expression in high-risk signature (orange), whereas DDIT4 present higher in low-risk signature (blue). The distribution of hub genes was marked as low proportion (green) or high proportion (red), separated by the median (vertical black line). Each point presented their connection with overall survival (B), high- and low-risk score group(C). (D) Number at risk for each stratified risk group over 20 years is processed using Kaplan-Meier method. (E) The efficacy of risk score in predicting overall survival through ROC curve and the AUC is 0.732.

**Supplementary Figure 4.** Discussing the efficacy of different reported models regarding prognosis-based GRGs in the ROC curve. The AUC of our GRG signature (GRGSig) is 0.73, which was superior than the previous studies in HNSCC, namely the signatures of Chen’s study (ChenSig, AUC = 0.51) and Liu’s study (LiuSig, AUC = 0.56).
