# Supplementary material for: Glycolysis related gene expression signature in predicting prognosis of laryngeal squamous cell carcinoma
Source: Bioengineered. 2021 Oct 29;12(1):8738–52. doi: 10.1080/21655979.2021.1980177 (PMC8806568; doi:10.1080/21655979.2021.1980177)
Supplement: Supplemental Material [file KBIE_A_1980177_SM4054.zip › supplementary/Supplementary Table 2 (1).docx]

**Supplementary Table 2. Clinical information of 17 male patients with LSCC**

| **Characteristics** | **Amount(proportion)** |
| --- | --- |
| Age (>60y/ ≤60y) | 15(88.2%)/ 2(11.8%) |
| HTN (No/ Yes) | 11(64.7%)/6(35.3%) |
| DM (No/ Yes) | 17(100%)/ 0 |
| Smoking history (No/ Yes) | 6(35.3%)/11(64.7%) |
| Drinking history (No/ Yes) | 12(70.6%)/5(29.4%) |
| Tumor location (Glottis/ Supraglottis/ Subglottis) | 12(70.6.8%)/ 4(23.5%)/ 1(5.9%) |
| TNM Stage (II/ III/ IV) | 3(17.6%)/ 8(47.1%)/ 6(35.3%) |
| T classification (T2/ T3/ T4) | 5(29.4%)/ 6(35.3%)/ 6(35.3%) |
| N classification (N0/ N+) | 13(76.5%)/ 4(23.5%) |
| Abbreviation: HTN, hypertension; DM, diabetes mellitus | |
