# Supplementary material for: Glycolysis related gene expression signature in predicting prognosis of laryngeal squamous cell carcinoma
Source: Bioengineered. 2021 Oct 29;12(1):8738–52. doi: 10.1080/21655979.2021.1980177 (PMC8806568; doi:10.1080/21655979.2021.1980177)
Supplement: Supplemental Material [file KBIE_A_1980177_SM4054.zip › supplementary/Supplementary Table1 (1).docx]

**Supplementary Table 1. Clinical information of TCGA cohort**

| **Characteristics** | **Amount(proportion)** |
| --- | --- |
| Age (>60y/ ≤60y) | 47(42.3%)/ 64(57.7%) |
| Gender (Male/ Female) | 91(82%)/ 20(18%) |
| Grade (G1-G2/ G3) | 82(73.9%)/ 29(26.1%) |
| Status (Alive/ Dead) | 61(55%)/ 50(45%) |
| TNM Stage (I-II/III-IV/ Not available) | 12(10.8%)/ 86(77.5%)/ 13(11.7%) |
| T classification (T1-2/ T3-4/ Not available) | 20(18%)/ 78(70.3%)/ 13(11.7%) |
| N classification (N0/ N+/ Not available) | 43(38.7%)/ 55(49.5%)/ 13(11.7%) |
| Abbreviation: Grade means pathological grade | |
