# Supplementary figures and images for: Glycolysis related gene expression signature in predicting prognosis of laryngeal squamous cell carcinoma
Source: Bioengineered. 2021 Oct 29;12(1):8738–52. doi: 10.1080/21655979.2021.1980177 (PMC8806568; doi:10.1080/21655979.2021.1980177)

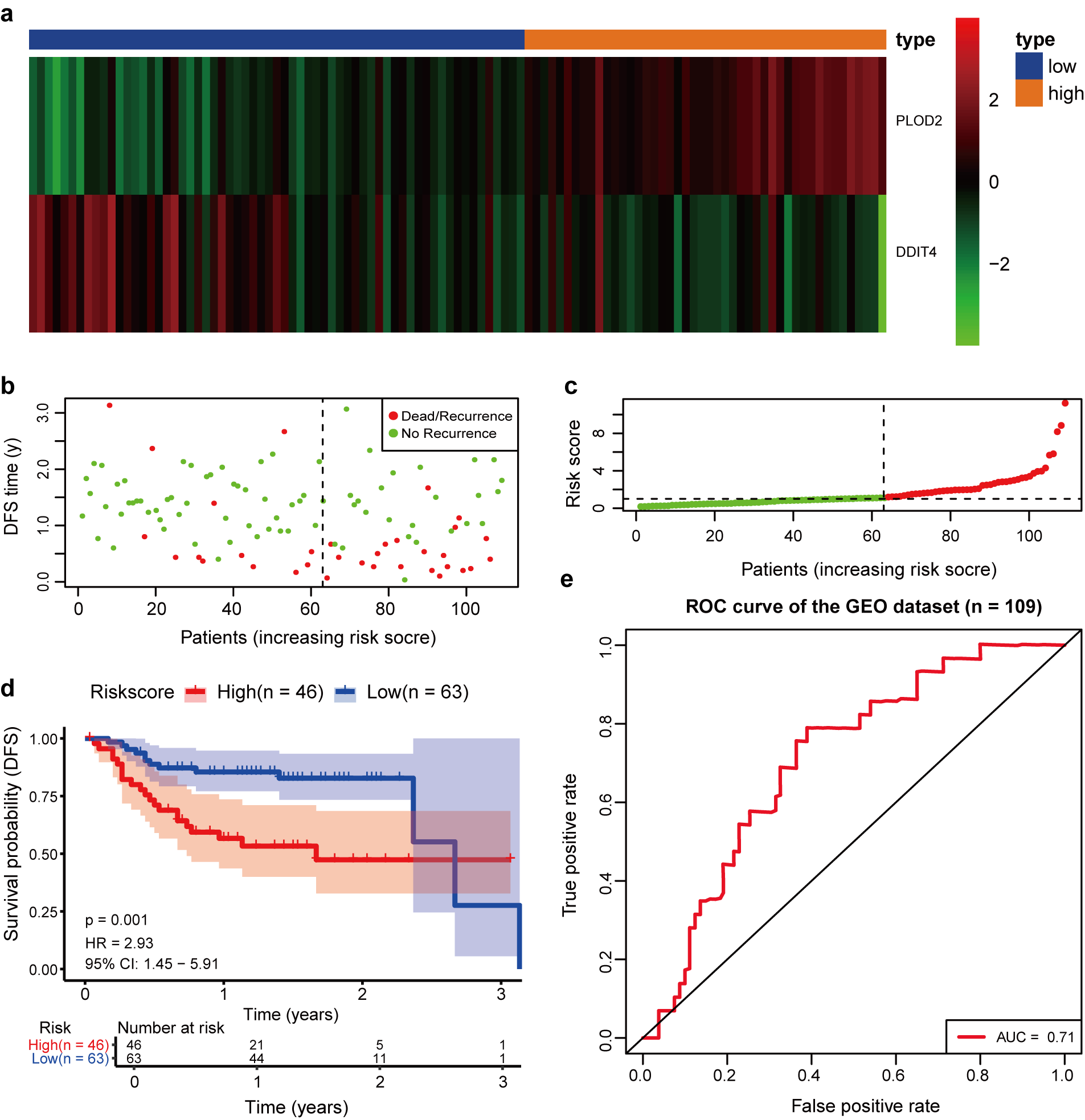

Supplement: Supplemental Material [file KBIE_A_1980177_SM4054.zip › supplementary/Supplementary Figure 1.png]

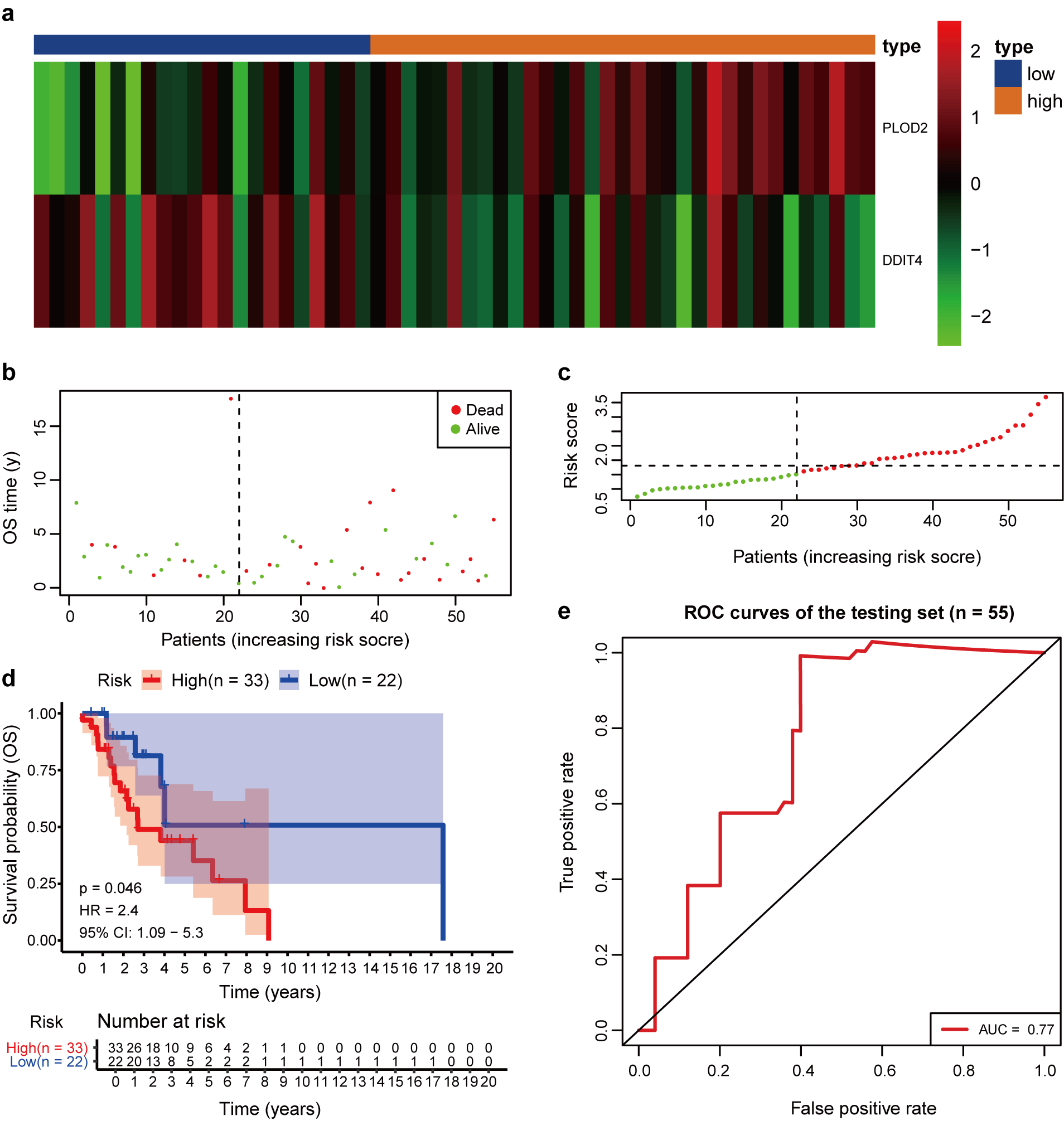

Supplement: Supplemental Material [file KBIE_A_1980177_SM4054.zip › supplementary/Supplementary Figure 2.png]

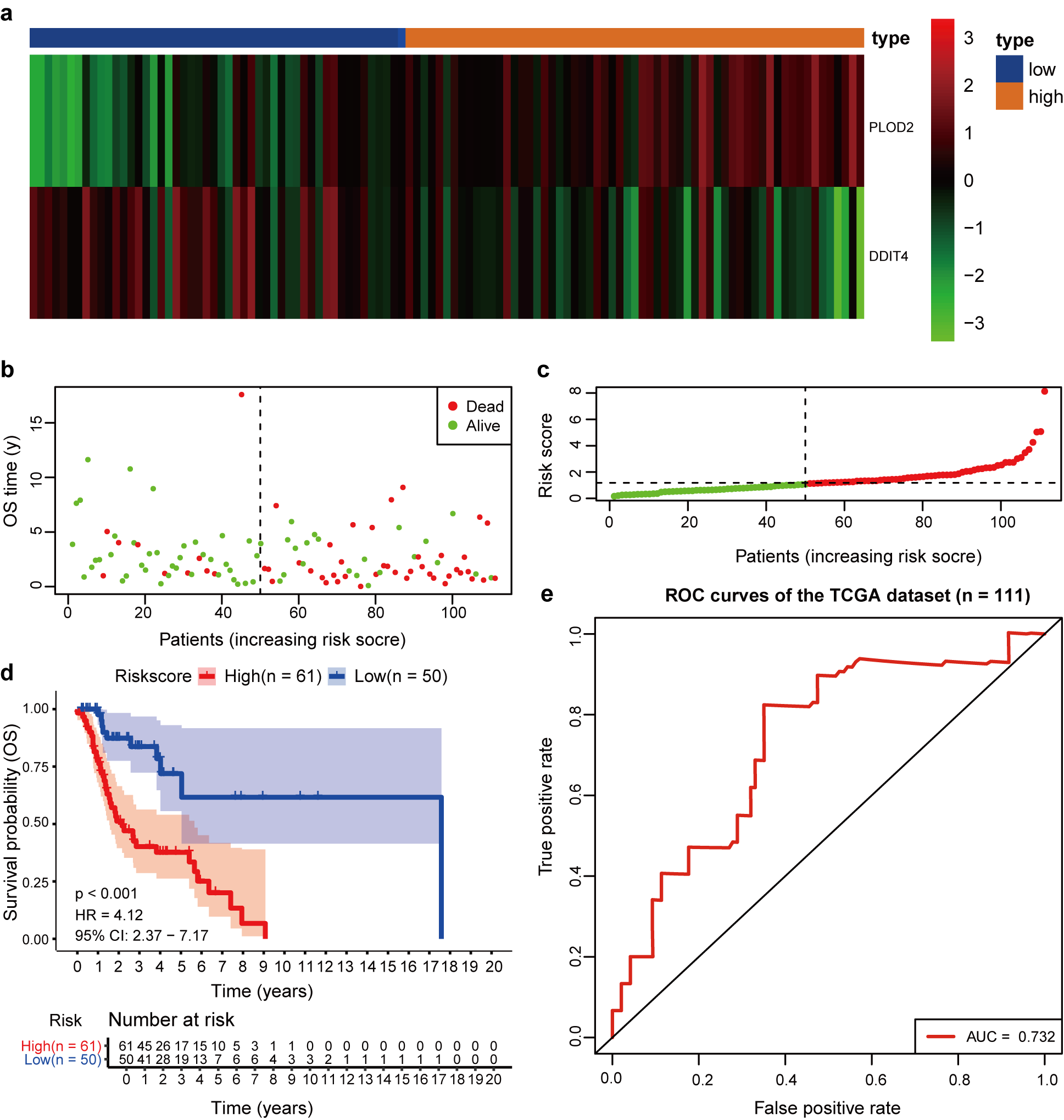

Supplement: Supplemental Material [file KBIE_A_1980177_SM4054.zip › supplementary/Supplementary Figure 3.png]

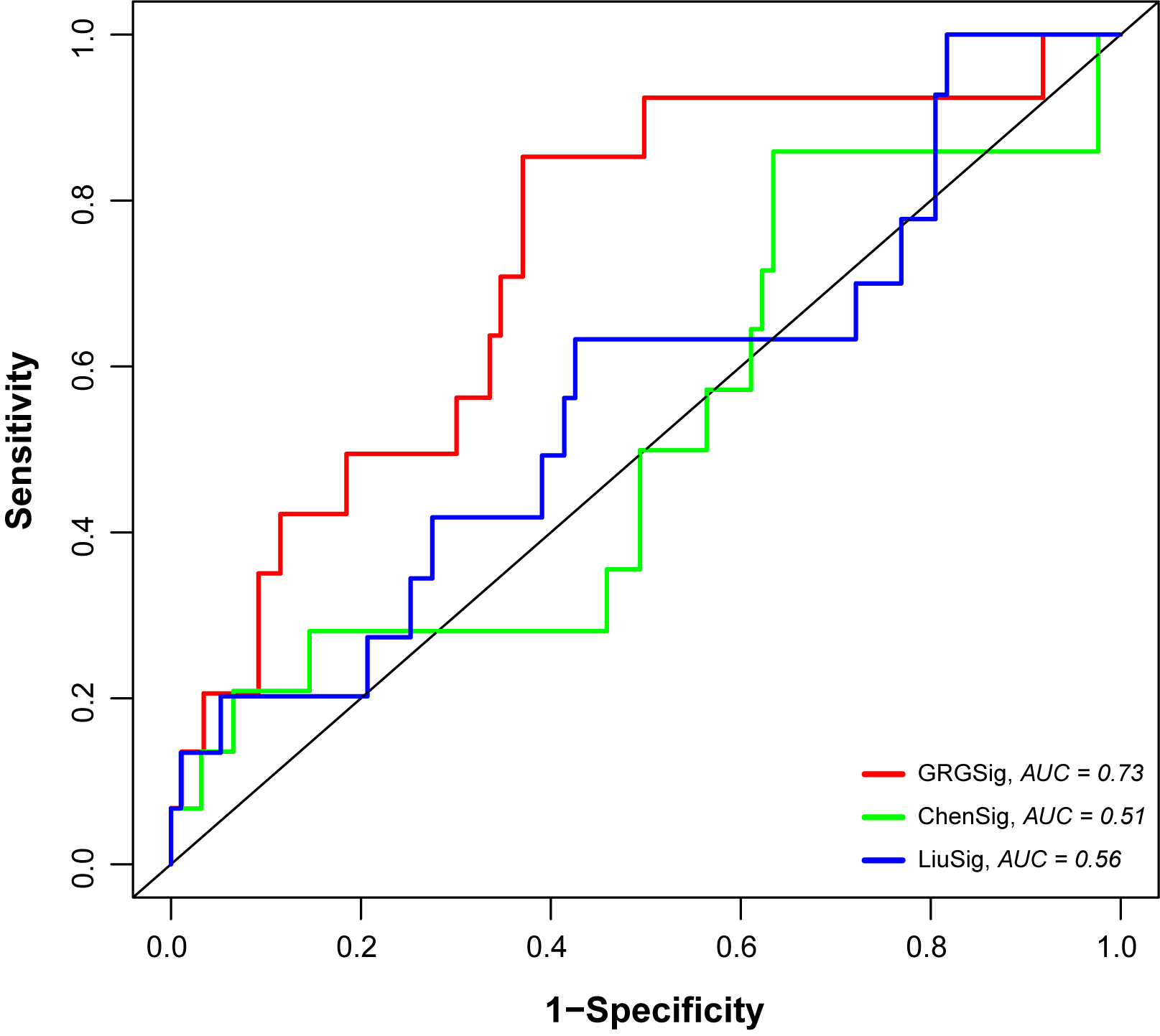

Supplement: Supplemental Material [file KBIE_A_1980177_SM4054.zip › supplementary/Supplementary Figure 4.png]
